# Is obesity associated with taste alterations? a systematic review

**DOI:** 10.3389/fendo.2023.1167119

**Published:** 2023-06-02

**Authors:** Beatriz Rodrigues Risuenho Peinado, Deborah Ribeiro Frazão, Leonardo Oliveira Bittencourt, Renata Duarte de Souza-Rodrigues, Maria Tereza Campos Vidigal, Douglas Teixeira da Silva, Luiz Renato Paranhos, Marcela Baraúna Magno, Nathalia Carolina Fernandes Fagundes, Lucianne Cople Maia, Rafael Rodrigues Lima

**Affiliations:** ^1^ Laboratory of Functional and Structural Biology, Institute of Biological Sciences, Universidade Federal do Pará, Belém, Brazil; ^2^ Division of Preventive and Community Dentistry, School of Dentistry, Federal University of Uberlandia, Uberlandia, Minas, Gerais, Brazil; ^3^ Department of Pediatric Dentistry and Orthodontics, School of Dentistry, Universidade Federal do Rio de Janeiro, Rio de Janeiro, Brazil; ^4^ School of Dentistry, Faculty of Medicine and Dentistry, University of Alberta, Edmonton, AB, Canada

**Keywords:** humans, obesity, overweight, taste, taste perception

## Abstract

**Background:**

Obesity is a growing chronic public health problem. The causes of obesity are varied, but food consumption decisions play an important role, especially decisions about what foods to eat and how much to consume. Food consumption decisions are driven, in part, by individual taste perceptions, a fact that can influence eating behavior and, therefore, body mass.

**Methodology:**

The searches were conducted in the electronic databases PubMed, Web of Science, Scopus, Lilacs, and the grey literature (Google Scholar and Open Grey). The acronym PECO will be used, covering studies with adult humans (P) who have obesity (E) compared to adult humans without obesity (C), having as an outcome the presence of taste alterations (O). After searching, duplicates were removed. The articles were first evaluated by title and abstract, following the inclusion and exclusion criteria; then, the papers were read in full. After the studies were selected, two reviewers extracted the data and assessed the individual risk of bias and control statements for possible confounders and bias consideration. The narrative GRADE system performed the methodological quality assessment using the New Castle Ottawa qualifier and analysis of certainty of evidence.

**Results:**

A total of 3782 records were identified from the database search, of these 19 were considered eligible. Forty percent of the eligible studies show that there was an association between obesity and different taste alterations for different flavors comparing with normal weights adults. In the methodological quality analysis of the nineteen studies, which assesses the risk of bias in the results, fifteen showed good methodological reliability, three showed fair methodological reliability, and one showed low methodological reliability.

**Conclusion:**

Despite methodological limitations, the results of the studies suggest the existence of a association between obesity and taste alterations, but further investigations with more sensitive methodologies are necessary to confirm this hypothesis.

**Systematic review registration:**

https://osf.io/9vg4h/, identifier 9vg4h.

## 1 Introduction

Obesity is a growing public health problem (1), since according to the World Obesity Atlas 2022 estimate published by the World Obesity Federation, the world will have, on average, one billion obese people in 2030. According to the latest global estimation from the World Health Organization (WHO), worldwide, obesity cases have more than doubled since 1980 (2).

An excessive increase in body fat characterizes this disease. It is associated with several deleterious aspects, such as lack of physical activity, poor eating habits, genetic conditions (e.g., mutations in the leptin gene or receptor), central nervous system abnormalities (e.g., hypothalamic alterations), hormonal factors (e.g., resistance to insulin action), psychological disorders (e.g., depression and anxiety), and socioeconomic factors (e.g., purchasing power) and obesity at levels directly proportional (3–9).

According to the WHO, the diagnosis of obesity based on is the parameter stipulated by the World Health Organization - the body mass index (BMI), obtained from the relationship between body weight (kg) and height (m)² of individuals. An individual is classified as obese when your BMI (Body Mass Index) exceeds 30kg/m². In addition, obesity has three levels of classification: Obesity Grade I with a BMI between 30 and 34.9 kg/m², Grade II obesity with a BMI between 35 and 39.9 kg/m² and Grade III obesity from 40 kg/m², also known as obesity morbid (2).

Much of the investigation on obesity is focused on individuals’ eating behavior or food reward response rather than the sensory aspects of eating, so the complex link between taste perception and BMI is unclear (10). The decisions of which and how much food to consume have significant relevance in the issue of body weight gain and these decisions are promoted, in part, by the taste perceptions of each individual (1).

Taste perception, provided mainly by the taste buds present at the beginning of the digestive tract, is directly associated with food intake. Therefore, alterations in this perception can interfere with a healthy eating routine and lead to problems such as ingesting toxic products (for example, spoiled or poisoned foods) or the overconsumption of certain foods (11). Two-thirds of the taste buds are located on the tongue and the rest are in the epiglottic vallecula and soft palate (12).

A critical hypothesis was formulated by Cabanac, theorizing the existence of a homeostatic set point of body weight inherent and individualized to each individual. This theory is defended through the principle of alaesthesia, characterized as a phenomenon in which, the first amount of food or substance causes a palatable pleasant sensation, however, as the consumption of the same food or substance continues and increases, an unpleasant sensation begins to be felt, which resembles the conception of specific sensory satiety. The authors say that alaesthesia depends on as yet unknown internal signals and suggest that this signal may be the variation in systemic glucose concentration (13, 14).

Altered taste perception can also lead to unbalanced eating routines, leading to over-nutrition or malnutrition resulting in disease conditions such as cognitive deficits, sensory deficits, immunity problems, malnutrition, cardiovascular problems, and neurodegeneration caused by a chronic high-fat diet. Therefore, investigations must be carried out on the sensory aspects of food intake, that is, sensitivity, preference, intensity of perception and specific sensory sensitivity, to understand the link between taste perception and obesity (2, 15–18).

The role of taste factors in obesity is a clinically important issue, as dietary non-compliance is a major issue in managing obesity and associated diseases such as diabetes. Therefore, quantifying the differences in taste perception may represent a new risk factor for obesity or obesity phenotype and inform future weight loss interventions. So far, no records in the literature of any previous systematic review on this subject exist. Thus, this systematic review aims to assess the association between obesity and taste alterations in adults.

## 2 Methods

### 2.1 Protocol and registration

The protocol was reported in accordance with the Preferred Reporting Items for Systematic Review and Meta-Analysis Protocols (PRISMA-P) (19) and is available in the Open Science Framework (OSF) database at the following link: (osf.io/9vg4h). This systematic review was reported in accordance with the Preferred Reporting Items for Systematic Reviews and Meta-Analyses (PRISMA) (20).

### 2.2 Selection criteria

The review was designed to answer whether there is an association between obesity and taste alterations in adult patients. The PECO strategy was used to define the eligibility criteria, where “P” represents the population (adult patients), “E” the exposure (obesity), “C” the comparison (subjects without obesity) and “O” the outcome (taste alteration).

The inclusion criteria for selection of articles were as follows (1): prospective or retrospective observational studies as cross-sectional, case-control, or cohort studies being conducted in adult humans with no others systemic diseases, and (2) studies whose focus was the comparison of taste sensitivity in average weight and obese subjects. There were no restrictions on the language or year of publication.

We excluded studies with sample overlapping (in this case, considering the most recent study that best described the methodology and results); Studies being conducted in animal, children or teenagers and *in vitro* studies; Case reports, reviews, descriptive studies, opinion articles, technical articles, editorials, letters to the editor, personal opinions, books, and book chapters.

### 2.3 Search strategy

Two authors (BRRP and DRF) used a search strategy consisting of MeSH and free terms to systematically search the online databases: PubMed, Scopus, Web of Science, Lilacs, and the grey literature (Google Scholar and Open Grey). The primary descriptors used to compose the search strategies were: “Obesity”; “Taste”; “Adult”. Several combinations among the descriptors were performed with the Boolean operators AND and OR, respecting the syntax rules of each database. The search strategy containing the keywords used in the search bases are included in the **Table 1**. These procedures were taken to reduce selection bias. Until December 2022, all databases’ searches were regularly updated.

**Table 1:** Terms used on databases searches.

Databases	Search strategy
**Pubmed**	#1 (((((((((Humans[MeSH Terms]) OR Humans[Title/Abstract]) OR Homo sapiens[Title/Abstract]) OR Man (Taxonomy)[Title/Abstract]) OR Man, Modern[Title/Abstract]) OR Modern Man[Title/Abstract]) OR Human[Title/Abstract]) OR Adult[MeSH Terms]) OR Adult[Title/Abstract]) OR Adults[Title/Abstract] 11.1.1 #2 (((((((((((((((((((((((((((((((((((((((((((((Obesity[MeSH Terms]) OR Obesity[Title/Abstract]) OR Overweight[MeSH Terms]) OR Overweight[Title/Abstract]) OR Overnutrition[MeSH Terms]) OR Overnutrition[Title/Abstract]) OR Hypernutrition[Title/Abstract]) OR Obesity, Abdominal[MeSH Terms]) OR Obesity, Abdominal[Title/Abstract]) OR Abdominal Obesities[Title/Abstract]) OR Obesities, Abdominal[Title/Abstract]) OR Abdominal Obesity[Title/Abstract]) OR Central Obesity[Title/Abstract]) OR Central Obesities[Title/Abstract]) OR Obesities, Central[Title/Abstract]) OR Obesity, Central[Title/Abstract]) OR Obesity, Visceral[Title/Abstract]) OR Visceral Obesity[Title/Abstract]) OR Obesities, Visceral[Title/Abstract]) OR Visceral Obesities[Title/Abstract]) OR Obesity, Metabolically Benign[MeSH Terms]) OR Obesity, Metabolically Benign[Title/Abstract]) OR Benign Obesity, Metabolically[Title/Abstract]) OR Metabolically Healthy Obesity[Title/Abstract]) OR Healthy Obesity, Metabolically [Title/Abstract]) OR Obesity, Metabolically Healthy[Title/Abstract]) OR Metabolically Benign Obesity[Title/Abstract]) OR Obesity, Morbid[MeSH Terms]) OR Obesity, Morbid[Title/Abstract]) OR Morbid Obesities[Title/Abstract]) OR Obesities, Morbid[Title/Abstract]) OR Obesity, Severe[Title/Abstract]) OR Obesities, Severe[Title/Abstract]) OR Severe Obesities[Title/Abstract]) OR Severe Obesity[Title/Abstract]) OR Morbid Obesity[Title/Abstract]) OR Weight Gain[MeSH Terms]) OR Weight Gain[Title/Abstract]) OR Gain, Weight[Title/Abstract]) OR Gains, Weight[Title/Abstract]) OR Weight Gains[Title/Abstract]) OR Body Weight[MeSH Terms]) OR Body Weight[Title/Abstract]) OR Body Weights[Title/Abstract]) OR Weight, Body[Title/Abstract]) OR Weights, Body[Title/Abstract] #3 ((((((((((((((((((((Ideal Body Weight[MeSH Terms]) OR Ideal Body Weight[Title/Abstract]) OR Body Weight, Ideal[Title/Abstract]) OR Body Weights, Ideal[Title/Abstract]) OR Ideal Body Weights[Title/Abstract]) OR Weight, Ideal Body[Title/Abstract]) OR Weights, Ideal Body[Title/Abstract]) OR Normal Body Weight[Title/Abstract]) OR Body Weight, Normal[Title/Abstract]) OR Body Weights, Normal[Title/Abstract]) OR Normal Body Weights[Title/Abstract]) OR Weight, Normal Body[Title/Abstract]) OR Weights, Normal Body[Title/Abstract]) OR Ideal Body Mass[Title/Abstract]) OR Body Mass, Ideal[Title/Abstract]) OR Body Masses, Ideal[Title/Abstract]) OR Ideal Body Masses[Title/Abstract]) OR Mass, Ideal Body[Title/Abstract]) OR Masses, Ideal Body[Title/Abstract]) OR Ideal Body Weight Formula[Title/Abstract]) OR Ideal Body Weight Chart [Title/Abstract] #4 ((((((((((((((((((((((Taste[MeSH Terms]) OR Taste[Title/Abstract]) OR Tastes[Title/Abstract]) OR Taste Sense[Title/Abstract]) OR Sense, Taste[Title/Abstract]) OR Senses, Taste[Title/Abstract]) OR Taste Senses[Title/Abstract]) OR Gustation[Title/Abstract]) OR Gustations[Title/Abstract]) OR Taste Perception[MeSH Terms]) OR Taste Perception[Title/Abstract]) OR Perception, Taste[Title/Abstract]) OR Perceptions, Taste[Title/Abstract]) OR Taste Perceptions[Title/Abstract]) OR Gustatory Perception [Title/Abstract]) OR Gustatory Perceptions[Title/Abstract]) OR Perception, Gustatory[Title/Abstract]) OR Perceptions, Gustatory[Title/Abstract]) OR Taste Threshold[MeSH Terms]) OR Taste Threshold[Title/Abstract]) OR Taste Thresholds[Title/Abstract]) OR Threshold, Taste [Title/Abstract]) OR Thresholds, Taste[Title/Abstract]
**Scopus**	#1 (TITLE-ABS-KEY (humans) OR TITLE-ABS-KEY (“Homo sapiens”) OR TITLE-ABS-KEY (“Man (Taxonomy)”) OR TITLE-ABS-KEY (“Man, Modern”) OR TITLE-ABS-KEY (“Modern Man”) OR TITLE-ABS-KEY (human) OR TITLE-ABS-KEY (adult) OR TITLE-ABS-KEY (adults)) #2 11.2 (TITLE-ABS-KEY (*obesity*) OR TITLE-ABS-KEY (*overweight*) OR TITLE-ABS-KEY (*overnutrition*) OR TITLE-ABS-KEY (*hypernutrition*) OR TITLE-ABS-KEY (*“Obesity, Abdominal”*) OR TITLE-ABS-KEY (*“Abdominal Obesities”*) OR TITLE-ABS-KEY (*“Obesities, Abdominal”*) OR TITLE-ABS-KEY (*“Abdominal Obesity”*) OR TITLE-ABS-KEY (*“Central Obesity”*) OR TITLE-ABS-KEY (*“Central Obesities”*) OR TITLE-ABS-KEY (*“Obesities, Central”*) OR TITLE-ABS-KEY (*“Obesity, Central”*) OR TITLE-ABS-KEY (*“Obesity, Visceral”*) OR TITLE-ABS-KEY (*“Visceral Obesity”*) OR TITLE-ABS-KEY (*“Obesities, Visceral”*) OR TITLE-ABS-KEY (*“Visceral Obesities”*) OR TITLE-ABS-KEY (*“Obesity, Metabolically Benign”*) OR TITLE-ABS-KEY (*“Benign Obesity, Metabolically”*) OR TITLE-ABS-KEY (*“Metabolically Healthy Obesity”*) OR TITLE-ABS-KEY (*“Healthy Obesity, Metabolically”*) OR TITLE-ABS-KEY (*“Obesity, Metabolically Healthy”*) OR TITLE-ABS-KEY (*“Metabolically Benign Obesity”*) OR TITLE-ABS-KEY (*“Obesity, Morbid”*) OR TITLE-ABS-KEY (*“Morbid Obesities”*) OR TITLE-ABS-KEY (*“Obesities, Morbid”*) OR TITLE-ABS-KEY (*“Obesity, Severe”*) OR TITLE-ABS-KEY (*“Obesities, Severe”*) OR TITLE-ABS-KEY (*“Severe Obesities”*) OR TITLE-ABS-KEY (*“Severe Obesity”*) OR TITLE-ABS-KEY (*“Morbid Obesity”*)) #2’1 11.3 (TITLE-ABS-KEY (*“Weight Gain”*) OR TITLE-ABS-KEY (*“Gain, Weight”*) OR TITLE-ABS-KEY (*“Gains, Weight”*) OR TITLE-ABS-KEY (*“Weight Gains”*) OR TITLE-ABS-KEY (*“Body Weight”*) OR TITLE-ABS-KEY (*“Body Weights”*) OR TITLE-ABS-KEY (*“Weight, Body”*) OR TITLE-ABS-KEY (*“Weights, Body”*)) #3 11.4 (TITLE-ABS-KEY (*“Ideal Body Weight”*) OR TITLE-ABS-KEY (*“Body Weight, Ideal”*) OR TITLE-ABS-KEY (*“Body Weights, Ideal”*) OR TITLE-ABS-KEY (*“Ideal Body Weights”*) OR TITLE-ABS-KEY (*“Weight, Ideal Body”*) OR TITLE-ABS-KEY (*“Weights, Ideal Body”*) OR TITLE-ABS-KEY (*“Normal Body Weight”*) OR TITLE-ABS-KEY (*“Body Weight, Normal”*) OR TITLE-ABS-KEY (*“Body Weights, Normal”*) OR TITLE-ABS-KEY (*“Normal Body Weights”*) OR TITLE-ABS-KEY (*“Weight, Normal Body”*) OR TITLE-ABS-KEY (*“Weights, Normal Body”*) OR TITLE-ABS-KEY (*“Ideal Body Mass”*) OR TITLE-ABS-KEY (*“Body Mass, Ideal”*) OR TITLE-ABS-KEY (*“Body Masses, Ideal”*) OR TITLE-ABS-KEY (*“Ideal Body Masses”*) OR TITLE-ABS-KEY (*“Mass, Ideal Body”*) OR TITLE-ABS-KEY (*“Masses, Ideal Body”*) OR TITLE-ABS-KEY (*“Ideal Body Weight Formula”*) OR TITLE-ABS-KEY (*“Ideal Body Weight Chart”*)) #4 11.5 (TITLE-ABS-KEY (*taste*) OR TITLE-ABS-KEY (*tastes*) OR TITLE-ABS-KEY (*“Taste Sense”*) OR TITLE-ABS-KEY (*“Sense, Taste”*) OR TITLE-ABS-KEY (*“Senses, Taste”*) OR TITLE-ABS-KEY (*“Taste Senses”*) OR TITLE-ABS-KEY (*gustation*) OR TITLE-ABS-KEY (*gustations*) OR TITLE-ABS-KEY (*“Taste Perception”*) OR TITLE-ABS-KEY (*“Perception, Taste”*) OR TITLE-ABS-KEY (*“Perceptions, Taste”*) OR TITLE-ABS-KEY (*“Taste Perceptions”*) OR TITLE-ABS-KEY (*“Gustatory Perception”*) OR TITLE-ABS-KEY (*“Gustatory Perceptions”*) OR TITLE-ABS-KEY (*“Perception, Gustatory”*) OR TITLE-ABS-KEY (*“Perceptions, Gustatory”*) OR TITLE-ABS-KEY (*“Taste Threshold”*) OR TITLE-ABS-KEY (*“Taste Thresholds”*) OR TITLE-ABS-KEY (*“Threshold, Taste”*) OR TITLE-ABS-KEY (*“Thresholds, Taste”*))
**Web of science**	#1 TÓPICO: (Human*) *OR* TÓPICO: (“Homo sapiens”) *OR* TÓPICO: (“Man (Taxonomy)”) *OR* TÓPICO: (“Man, Modern”) *OR* TÓPICO: (“Modern Man”) *OR* TÓPICO: (Adult*) #2 TÓPICO: (Obesity) *OR* TÓPICO: (Overweight) *OR* TÓPICO: (Overnutrition) *OR* TÓPICO: (Hypernutrition) *OR* TÓPICO: (“Modern Man”) *OR* TÓPICO: (“Obesit*, Abdominal”) *OR* TÓPICO: (“Abdominal Obesit*”) *OR* TÓPICO: (“Central Obesit*”) *OR* TÓPICO: (“Obesit*, Central”) *OR* TÓPICO: (“Obesit*, Visceral”) *OR* TÓPICO: (“Visceral Obesit*”) *OR* TÓPICO: (“Obesity, Metabolically Benign”) *OR* TÓPICO: (“Benign Obesity, Metabolically”) *OR* TÓPICO: (“Metabolically Healthy Obesity”) *OR* TÓPICO: (“Healthy Obesity, Metabolically”) *OR* TÓPICO: (“Obesity, Metabolically Healthy”) *OR* TÓPICO: (“Metabolically Benign Obesity”) *OR* TÓPICO: (“Obesit*, Morbid”) *OR* TÓPICO: (“Morbid Obesit*”) *OR* TÓPICO: (“Obesit*, Severe”) *OR* TÓPICO: (“Severe Obesit*”) *OR* TÓPICO: (“Weight Gain*”) *OR* TÓPICO: (“Gain*, Weight”) *OR* TÓPICO: (“Body Weight*”) *OR* TÓPICO: (“Weight*, Body”) #3 TÓPICO: (“Ideal Body Weight*”) *OR* TÓPICO: (“Body Weight*, Ideal”) *OR* TÓPICO: (“Weight*, Ideal Body”) *OR* TÓPICO: (“Normal Body Weight*”) *OR* TÓPICO: (“Body Weight*, Normal”) *OR* TÓPICO: (“Weight*, Normal Body”) *OR* TÓPICO: (“Ideal Body Mass*”) *OR* TÓPICO: (“Body Mass*, Ideal”) *OR* TÓPICO: (“Mass*, Ideal Body”) *OR* TÓPICO: (“Ideal Body Weight Formula”) *OR* TÓPICO: (“Ideal Body Weight Chart”) #4 TÓPICO: (Taste*) *OR* TÓPICO: (“Taste Sense*”) *OR* TÓPICO: (“Sense*, Taste”) *OR* TÓPICO: (Gustation*) *OR* TÓPICO: (“Taste Perception*”) *OR* TÓPICO: (“Perception*, Taste”) *OR* TÓPICO: (“Gustatory Perception*”) *OR* TÓPICO: (“Perception*, Gustatory”) *OR* TÓPICO: (“Taste Threshold*”) *OR* TÓPICO: (“Threshold*, Taste”)
**Cochrane**	#1 Humans OR Human OR “Homo sapiens” OR “Man (Taxonomy)” OR “Man, Modern” OR “Modern Man” OR Adult OR Adults #2 Obesity OR Overweight OR Overnutrition OR Hypernutrition OR “Obesity, Abdominal” OR “Obesities, Abdominal” OR “Abdominal Obesities” OR “Abdominal Obesity” OR “Central Obesity” OR “Central Obesities” OR “Obesities, Central” OR “Obesity, Central” OR “Obesity, Visceral” OR “Obesities, Visceral” OR “Obesity, Metabolically Benign” OR “Benign Obesity, Metabolically” OR “Metabolically Healthy Obesity” OR “Healthy Obesity, Metabolically” OR “Obesity, Metabolically Healthy” OR “Metabolically Benign Obesity” OR “Obesity, Morbid” OR “Obesities, Morbid” OR “Morbid Obesities” OR “Obesity, Severe” OR “Obesities, Severe” OR “Severe Obesities” OR “Severe Obesity” OR “Morbid Obesity” OR “Weight Gain” OR “Gain, Weight” OR “Gains, Weight” OR “Weight Gains” OR “Body Weight” OR “Body Weights” OR “Weight, Body” OR “Weights, Body” #3 “Ideal Body Weight” OR “Body Weight, Ideal” OR “Body Weights, Ideal” OR “Ideal Body Weights” OR “Weight, Ideal Body” OR “Weights, Ideal Body” OR “Normal Body Weight” OR “Body Weight, Normal” OR “Body Weights, Normal” OR “Normal Body Weights” OR “Weight, Normal Body” OR “Weights, Normal Body” OR “Ideal Body Mass” OR “Body Mass, Ideal” OR “Body Masses, Ideal” OR “Ideal Body Masses” OR “Mass, Ideal Body” OR “Masses, Ideal Body” OR “Ideal Body Weight Formula” OR “Ideal Body Weight Chart” #4 Taste OR Tastes OR “Taste Sense” OR “Sense, Taste” OR “Senses, Taste” OR “Taste Senses” OR Gustation OR Gustations OR “Taste Perception” OR “Perception, Taste” OR “Perceptions, Taste” OR “Taste Perceptions” OR “Gustatory Perception” OR “Gustatory Perceptions” OR “Perception, Gustatory” OR “Perceptions, Gustatory” OR “Taste Threshold” OR “Taste Thresholds” OR “Threshold, Taste” OR “Thresholds, Taste”
**Lilacs**	(tw:(Humans OR Human OR (“Homo sapiens”) OR (“Man (Taxonomy)”) OR (“Man, Modern”) OR (“Modern Man”) OR Adult OR Adults)) AND (tw:(Obesity OR Overweight OR Overnutrition OR Hypernutrition OR (“Obesity, Abdominal”) OR (“Obesities, Abdominal”) OR (“Abdominal Obesities”) OR (“Abdominal Obesity”) OR (“Central Obesity”) OR (“Central Obesities”) OR (“Obesities, Central”) OR (“Obesity, Central”) OR (“Obesity, Visceral”) OR (“Obesities, Visceral”) OR (“visceral obesity”) OR (“visceral obesities”) OR (“Obesity, Metabolically Benign”) OR (“Benign Obesity, Metabolically”) OR (“Metabolically Healthy Obesity”) OR (“Healthy Obesity, Metabolically”) OR (“Obesity, Metabolically Healthy”) OR (“Metabolically Benign Obesity”) OR (“Obesity, Morbid”) OR (“Obesities, Morbid”) OR (“Morbid Obesities”) OR (“Obesity, Severe”) OR (“Obesities, Severe”) OR (“Severe Obesities”) OR (“Severe Obesity”) OR (“Morbid Obesity”) OR (“Weight Gain”) OR (“Gain, Weight”) OR (“Gains, Weight”) OR (“Weight Gains”) OR (“Body Weight”) OR (“Body Weights”) OR (“Weight, Body”) OR (“Weights, Body”))) AND (tw:((“Ideal Body Weight”) OR (“Body Weight, Ideal”) OR (“Body Weights, Ideal”) OR (“Ideal Body Weights”) OR (“Weight, Ideal Body”) OR (“Weights, Ideal Body”) OR (“Normal Body Weight”) OR (“Body Weight, Normal”) OR (“Body Weights, Normal”) OR (“Normal Body Weights”) OR (“Weight, Normal Body”) OR (“Weights, Normal Body”) OR (“Ideal Body Mass”) OR (“Body Mass, Ideal”) OR (“Body Masses, Ideal”) OR (“Ideal Body Masses”) OR (“Mass, Ideal Body”) OR (“Masses, Ideal Body”) OR (“Ideal Body Weight Formula”) OR (“Ideal Body Weight Chart”))) AND (tw:(Taste OR Tastes OR (“Taste Sense”) OR (“Sense, Taste”) OR (“Senses, Taste”) OR (“Taste Senses”) OR Gustation OR Gustations OR (“Taste Perception”) OR (“Perception, Taste”) OR (“Perceptions, Taste”) OR (“Taste Perceptions”) OR (“Gustatory Perception”) OR (“Gustatory Perceptions”) OR (“Perception, Gustatory”) OR (“Perceptions, Gustatory”) OR (“Taste Threshold”) OR (“Taste Thresholds”) OR (“Threshold, Taste”) OR (“Thresholds, Taste”)))
**Opengrey**	Obesity AND Taste
**Google scholar**	Obesity AND “Taste Perception”

The recovered findings were transferred to the EndNote X9™ software (Clarivate™ Analytics, Philadelphia, USA), where duplicates were automatically deleted, and the remaining duplicates were manually removed. The gray literature was manually evaluated with Microsoft Word™ 2010 (Microsoft™ Ltd., Washington, USA) simultaneously and thoroughly.

### 2.4 Process of selecting studies

Prior to selecting the studies, two reviewers conducted a calibration exercise in which they reviewed the eligibility criteria and applied them to a sample of 20% of the retrieved studies to determine inter-examiner agreement. The selection process began once an appropriate degree of agreement (Kappa ≥ 0.81) was reached.

Two reviewers (BRRP and DRF) chose the studies after reading the titles and abstracts. A third examiner (LOB) interpreted and defined disagreements between the examiners. Subsequently, the preliminary eligible studies’ full texts were obtained and evaluated. If the full texts could not be located, a bibliographic request was made to the library database (COMUT), and an e-mail was sent to the corresponding authors to obtain the texts.

### 2.5 Data extraction

After a complete reading of all studies included, two reviewers (BRRP and DRF) independently and blindly extracted data from the eligible studies. When there was disagreement about data extraction, a third reviewer (LOB) looked into the issues. The following data were extracted: country, year, study design, main characteristics of the participants (origin, sample size and age), diagnosis of obesity, diagnosis of taste alterations, statistical analysis and results. For situations where relevant information was not available for data extraction and/or risk of bias analysis, the authors were contacted by email.

### 2.6 Risk of bias assessment

Two reviewers (BRRP and DRF) independently assessed the methodological quality/risk of bias of the studies included. In case of disagreement, a third reviewer (RRL) was consulted to make a final decision. The Newcastle-Ottawa Scale (NOS) (21) for assessing the quality of studies (Ottawa quality assessment scale case-control studies) was used. This scale consists of questions with predefined domains divided into selection, comparability, and exposure. The first section evaluates the study based on the case definition, the representativeness of the cases, and the selection and definition of the controls. The second domain assesses the comparability of cases and controls based on the design or analysis related to confounding factor control. The exposure section examines the method of determining exposure, the non-response rate, and whether the study used the same exposure assessment method for cases and controls. Thus, studies can get a maximum of nine “stars,” four “stars” for selection, two for comparability, and three for the outcome. These processes were repeated for each type of study included (cross-sectional, case-control, and cohort).

### 2.7 Data synthesis

Data extracted from articles fitting the inclusion criteria were analyzed using a narrative synthesis approach in systematic reviews consistent with best practices (22). Data as sample size and age were subgrouped into obese or normal weight. The way of diagnosing obesity was subgrouped into overweight or body mass index percentages. The perception threshold was tested with different flavors such as sweet, bitter, sour, salty and foods with a high-fat content to assess the taste alterations, so this could represent pleasant taste stimuli, sensitivity and preference. Ideally, a formal meta-analysis should be conducted to provide quantitative estimates of differences in taste perception between obese and normal-weight adults, but due to the heterogeneity in exposure metrics and methodologies used across eligible studies, a meta-analysis was not possible.

### 2.8 Certainty of evidence

The GRADE system (Grades of Recommendation, Assessment, Development and Evaluation) was used to assess the certainty of evidence. Four levels of reliability were assigned to the studies included: high, moderate, low, and very low. The highest level indicates strong confidence that the actual effect is close to that estimated, and the lowest level demonstrates that confidence in the effect estimate is very limited, with a significant degree of uncertainty in the findings.

## 3 Results

### 3.1 Study selection

**Figure 1:**
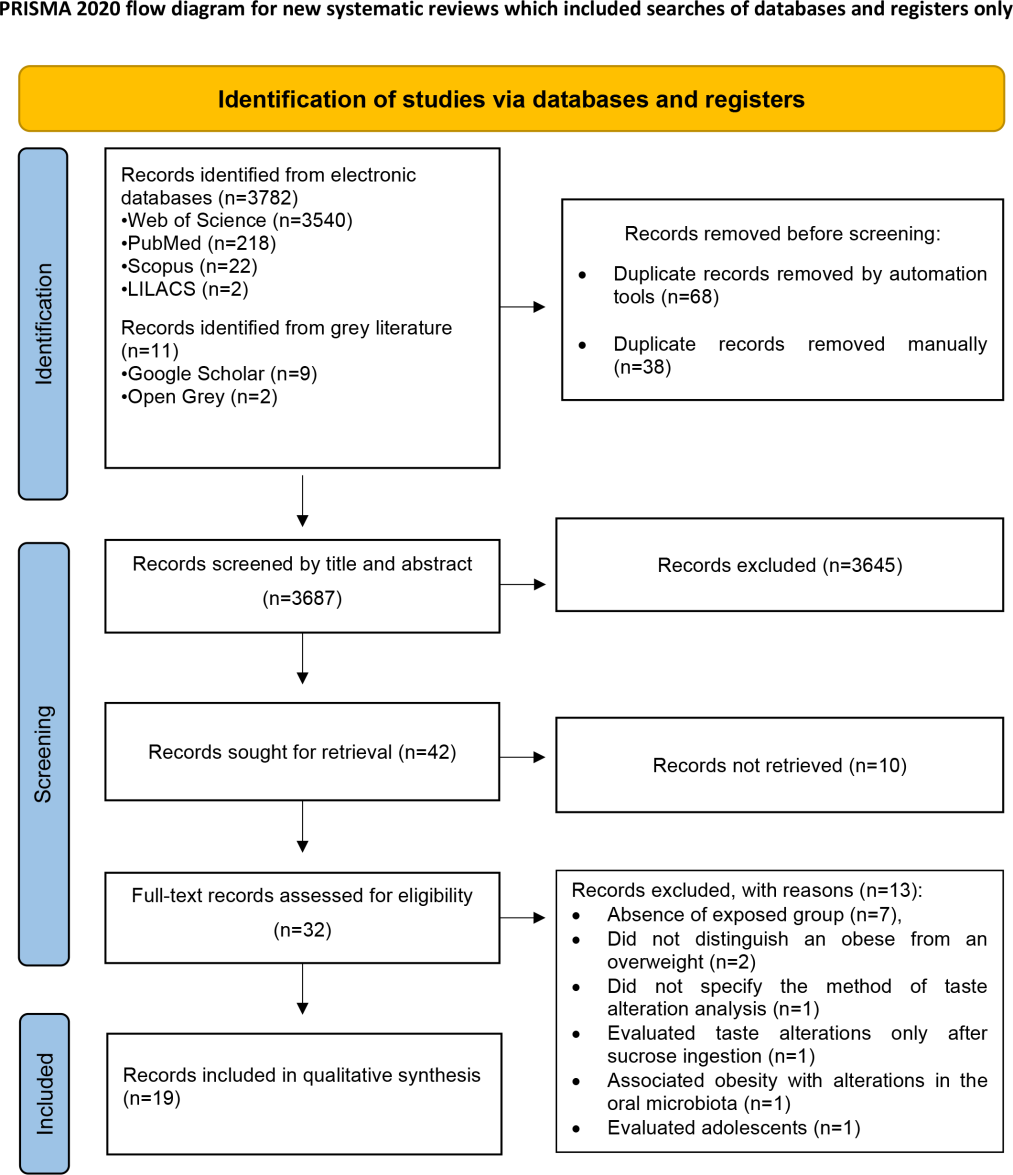
Flow diagram of databases searched according to PRISMA guidelines (Preferred Reporting Items for Systematic Review and Meta-Analysis).

Initially, 3793 records were found from the electronic databases, including the “grey literature.” Then, 106 duplicates were excluded, leaving 3687 articles. A careful reading of the titles and abstracts excluded 3645 studies, leaving 42 for full-text reading. However, 10 of them were not retrieved. In this way, the 32 articles retrieved were read in full. Finally, seven studies were excluded due to the absence of an exposed group, one for not specifying the method of analysis of the taste alteration, one for evaluating taste alterations only after sucrose ingestion, one for associating obesity with alterations in the oral microbiota, and two for the absence of separation between the obese and overweight groups. Thus, 19 articles were included in this review. **Figure 1** demonstrates the study selection process in detail.

### 3.2 Study characteristics

According to the study design, twelve are case-control studies, seven are cross-sectional studies, and one is a cohort study. The studies varied in different outcomes related to taste alterations and in various flavors analyzed: sweet, bitter, sour, salty, and foods with high-fat content.

Eight studies (12, 17, 18, 23–28) evaluated the perception threshold of the groups, that is, the smallest stimulus capable of provoking the least possible sensation of taste. Among the results obtained, six (12, 18, 24, 25, 27, 28) showed no difference between the groups for some flavors, and two (25, 26) concluded that people without obesity had a higher threshold of perception for sweet, salt, bitter and sour taste, and three (17, 18, 23, 24) studies showed that participants with obesity had a higher threshold compared to participants without obesity for same flavors, thus needing higher concentrations to taste any flavor.

Eight studies (11, 12, 14, 15, 24, 25, 29, 30) evaluated whether people with obesity had a reduced intensity of taste perception; that is, for the same supra-threshold concentration, a flavor can be felt less intensely by participants with obesity. Among these, no one obtained results that agreed with this hypothesis; seven results (11, 12, 14, 15, 24, 29, 30) did not identify this taste alteration in either of the two groups, one analysis (25) recognized that aspect in participants without obesity for sweet, salty and sour tastes. However, a specific article (5) investigated initial levels of perception between groups and the speed of decline of this perception with consumption. The result found was that individuals with obesity felt higher initial levels of taste perception than participants without obesity but experienced a slower rate of decline in this perception; that is, the exposed group felt the taste with the same intensity for a more extended period.

Regarding the outcome related to preference for higher concentrations, four results (3, 14, 15, 24) did not identify any difference between the groups evaluated regarding the concentration of sweetness and fat. In contrast, one result (14) found that the control group preferred lower concentrations of sweet fat, and two (25, 29) studies observed that the group with obesity preferred higher concentrations of sweet and one (24) for salty.

In the evaluations of the hedonic response presented by the participants, two (4, 11) studies found that both groups obtained the same level of hedonic response for sweet, salty, and bitter tastes, and one (2) study observed a more significant response of participants with obesity in relation to salty, sweet and fatty tastes.

Among the chosen articles, only two (7, 30) evaluated the outcome related to specific sensory satiety, which is basically defined as a decrease in pleasure and perception when food is consumed until satiety. Thus, one study (7) had similar results for both groups, and the other (30) observed that people with obesity take longer to feel this satiety.

The last analyzed outcome was the brain activation of the groups regarding food consumption, and the results of this study (13) showed that for sweet stimuli, participants with obesity showed greater brain activation in several areas related to gustatory perception. **Table 2** summarizes and presents more details of the included studies’ characteristics.

**Table 2:** Characteristics of included studies.

Author/Year/ Country	Study Design	Sample Size	Age range or mean (SD) in years	Obesity diagnosis	Taste changes evaluation	Outcomes
(31). Grinker et. al., 1972, United States	Case-control	Ob: 39 NW: 13	Ob: 21-51 NW: 17 - 27	Percentage of overweight	Sweet taste sensitivity	No differences in detectability were obtained between the obese and the normal weight subjects (nonpaired t test)
(32). Thompson et. al., 1976, United States	Case-control	Ob: 59 NW: 18	NA	BMI	Sweet taste pleasantness (hedonic response)	There was a clear inverse relationship between pleasantness response and obesity indices within each weight category. However, the discrepancies in the data do not rule out the possibility that changes in body fat percentage have an impact on hedonic response in both lean and obese subjects in a slow-acting, closed-loop, negative feedback system.
(33). Thompson et. al., 1977, United States	Case-control	Ob: 14 NW: 18	Ob and NW: 20 (3)	BMI	Sweet taste preference (greater concentration)	The geometric means of the sucrose satisfaction ratings increased to a maximum between concentrations of 0.15 and 0.6 M and then declined (hedonic response type I) or increased along with concentration (hedonic response type II) in individuals with normal weight and obese. Normal weight and obese individuals did not differ in intensity and hedonic ratings for sweet taste.
(27). Malcon, 1980, USA	Case-control	Ob: 7 NW: 7	Ob: 47.86 NW: 24.29	Percentage of overweight	Salty, sweet, sour and bitter taste thresholds levels and hedonic response	No group differences were observed on detection or recognition thresholds for any of the tastes. Analyses of rating of salty, sour and bitter revealed identical patterns of results. There were significant effects only of concentrations. For each of the three tastes, the entire sample showed a decline in rated pleasantness over increasing concentrations. Analysis of ratings of sweet showed no significant effects of groups, concentrations, or their interaction.
(29). Drewnowski et. al., 1985, United States	Case-control	Ob: 12 NW: 15	Ob and NW: 33.5 (1.7)	BMI	Sweet and fat taste sensitivity and preference (higher fat content)	Analysis of intensity ratings did not show significant differences between normal weight, obese and obese subjects with stable reduction. For individuals with normal weight, fatty stimuli were appreciated significantly more than equally sweet, but low-fat stimuli. Obese individuals enjoyed high-fat sugary stimuli as much as normal- weight individuals, but did not appreciate the equally sweet solutions of sucrose in fat-free milk
(34). Snoek et. al., 2004, Netherlands	Case-control	Ob: 21 NW: 23	Ob: 47 (11) NW 46 (10)	BMI	Sensitivity Hight and low fat foods (sweet and salty) sensory specific satiety	The results of this study do not confirm the hypothesis that there is a difference in sensory-specific satiety between obese and normal-weight subjects for products with high fat content. In fact, this study indicates that there is no difference in the degree of sensory-specific satiety for any of the foods tested between obese and normal-weight subjects.
(35). Pepino et. al., 2010, United States	Case-control	Ob: 23 NW 34	Ob and NW: 21 – 40	BMI	Umami and sweet taste sensitivity and preference (greater concentration)	Obese women had significantly higher detection thresholds for MSG (i.e., lower MSG taste sensitivity; F(1,54) = 4.90; P = 0.03), but not for sucrose (P = 0.84), than did normal-weight women. MSG and sucrose detection thresholds were not correlated (r(56df) = 0.19; P = 0.17). We found no statistical differences between the groups in the intensity of sucrose most preferred (P = 0.43), but obese women tended to prefer soups with higher MSG concentrations than did normal-weight women (F(1,55) = 3.28; P = 0.08). When MSG detection thresholds were included as a covariate in the analysis, this difference became statistically significant (F(1,53) = 5.44; P = 0.02)
(36). Vereczei, 2011, Hungary	Case-control	Ob: 10 NW: 10	NA	BMI	Brain activation to pleasant and unpleasant taste stimuli	When stimulated with 0.1 M cane sugar solution, the activity significantly higher was measured in the obese group compared to the control group in the caudolateral cortex and orbitofrontalis, the anterior cingulate gyrus, the amygdala, nucleus accumbens, putamen and caudatum. The use of 0.03 mM QHCl solution also resulted in significantly greater activation in the obesity group.
(37). Szalay, 2012, Hungary	Case-control	Ob: 12 NW: 12	Ob: 38.3 (4.2) NW: 37.1 (3.8)	BMI	Brain activation to pleasant taste stimuli	Significant differences were found between the two groups in the pleasantness ratings given for sucrose (62.5 ± 11.38 in obese vs. 27 ± 4.4 in controls; p<0.001), for quinine (± 9277.9 in obese vs. ± 67.5 ± 14.36 in controls; p<0.001), and for vanilla (94.5 ± 5.4 in obese vs. 48.75°111.89 in controls; p <0.001), respectively.
(38). Pepino and Mennella 2012, United States	Cross-sectional	Ob: 22 NW: 32	Ob and NW: 21 - 40	BMI	Sweet specific satiety, perception intensity and taste pleasantness	Obese women noticed the sweet tastes just as pleasant for a longer period of time (16 min) than lean women (p = 0.03). There were no statistical differences on the intensity of sucrose preferred by the two groups of women (p = 0.58). Both obese and lean women perceived the sweetness of the 24% w/v sucrose concentration equally intense across trials 1–10, 12 and 13 (p > 0.70). There were no statistical differences on the intensity of sucrose preferred by the two groups of women (p = 0.58). Lean women most preferred 16.1 ± 2.0% w/v sucrose solution, and obese women most preferred 17.9 ± 2.4% w/v sucrose solution. The change of hedonic value from positive to negative between trial 10 and 11 was significantly greater in obese than in lean women (lean: 3.1 ± 4.8; obese: 23.8 ± 5.8; F(1,52) = 7.44; p = 0.009)
(25). Deglaire et. al., 2015, France	Cohort	Ob: 4.993 NW: 29.263	Ob and NW Female: 47,7 (16,5) Male: 46,4 (16,2)	BMI	Sweet, salt and fat pleasantness	Overall liking scores for the salt, fat-and-salt and fat-and-sweet sensations were positively linearly associated with BMI in men and women (P≤0.05). The only scores for which there was no significant difference across BMI categories, that is no association, linear or not, with BMI, were for liking for sweet foods and for fatty-sweet foods in men and for liking for sweet in women.
(10). Tucker, 2014, United States	Cross-sectional	Ob: 11 NW: 24	NA	BMI	Bitter taste threshold	Threshold sensitivity to OA was measured in lean, overweight, and obese individuals over the course of 7 test visits. Statistical analyses revealed no differences between the lean and overweight group, so the 2 groups were combined (lean plus overweight). The slope of threshold concentrations versus visit was significantly different and negative for the lean plus overweight compared with the positive slope for the obese participants.
(39). Park et. al., 2015, Korea	Case-control	Ob: 18 NW: 23	Ob: 24.81 (2.45) NW: 23.68 (3.04)	BMI	Sweet, salty, sour and bitter taste thresholds	The results of the chemical taste tests revealed higher thresholds in the obese than in the normal weight group for sweet (0.70 n in t g/ml vs 0.33 (0.70 g/ml), salty (0.45 3 (0. g/ml vs 0.28 (0.70 g/ml), bitter (0.03 70 n i g/ml vs 0.01. 03 70 g/ml), and sour (0.22 3 70 n in l vs 0.18 ± 0.15 g/ml) tastes. However, only the threshold for salty taste was significantly higher in the obese than in the normal-weight group (p < 0.05)
(40). Proserpio et. al., 2016, Italy	Cross-sectional	Ob: 52 NW: 52	Ob and NW: 40.17 (10.79)	BMI	Bitter, sweet, salty, sour and fat taste thresholds	Significant differences between NW and OB subjects were found for all taste stimuli (sweet taste: df = 101, t = 3.48, P = 0.0002; salty taste: df = 101, t = 2.98, P = 0.003); bitter taste: df = 101, t = 3.00, P = 0.003; fat sensation: df = 101, t = 4.42, P = 0.00002, sour taste: df = 101, t = 2.15, P = 0.03). OB subjects showed higher thresholds values compared with NW controls
(2). Hardikar et. al., 2017, Germany	Case-control	Ob: 23 NW: 31	Ob and NW: 18 -35	BMI	Sweet, salty, sour and bitter threshold levels, perception intensity and sweet taste preference (higher fat content)	Obese (OB) had significantly lower thresholds for sweet and salty 219 (p = 0.003) than lean (LN). No significant group difference was found for sour and bitter. For the supra-threshold tastants, Obese tended to rate the “Absolute Low” and “Absolute High” concentrations as more intense than lean. This difference was significant for the “Absolute High” sweet (p=0.024), “Absolute Low” sweet (p=0.007), “Absolute Low” salty (p=0.01), and “Absolute Low” sour (p=0.004) concentrations. OB also rated the “Relative High” sweet (p=0.017) as more pleasant than the lean group.
(26). Karmous et. al., 2018, Tunisia	Case-control	Ob: 52 NW: 52	Ob: 35.3 (5.43) NW: 35.0 (4.10)	BMI	Bitter and fatty taste threshold levels and sensitivity	There were no difference in fatty detection thresholds between obese and control groups (p=0.18). Nonetheless, fatty oral sensitivity was associated with BMI in obese participants (p=0.037), but not in control subjects. The obese subjects exhibited higher PROP (bitter) detection threshold than normal weight subjects (p < 0.001).
(41). Fernandez - Garcia et. al., 2017, Spain	Cross sectional	Ob: 28 NW: 77	Ob and NW: 18 - 65	BMI	Sweet, salty, bitter and sour tastes thresholds levels	Regarding taste functions, most of the functions measured and the total taste strips (TS) correlated negatively with BMI. Sweet TS: r = -0.301, p < 0.001; Sour TS: r = -0.388, p < 0.001; Salt TS: r = -0.237, p = 0.002; Bitter TS: r = -0.239, p = 0.002; Total TS: r = -0.407, p < 0.001
(1). Miller et. al., 2019, United States	Case-control	Ob: 51 NW: 161	Ob: 44,7 NW: 35.2	BMI	Sweet taste perception and sensory specific satiety	Obese participants had higher levels of initial taste perception than lean subjects (P=0.02). Also, obese participants reported taste perceptions that declined slower than lean participants (P<0.01). For all groups, pretzels consumed at the beginning of the study provided the same taste perception as pretzels consumed at the end of the study, despite the substantial declines in hunger reported during the study period.
(30). Leohr et. al., 2020, United States	Cross-sectional	Ob: 23 NW: 24	Ob and NW: 26 - 45	BMI	Sweet perception and hedonic response	The perception of creaminess depends on fat and sugar content and is described by a proportional odds model with linear effects of sugar and fat. Enjoyment increases with sugar and fat and decreases with a 37.5% fat solution. Using a differential probability model for fat and sugar, there is a negative interaction between them, allowing for low-sugar and high-fat well-being.

NA, Not Available; BMI, Body Mass Index; Ob, Obese; NW, Normal Weight.

### 3.3 Risk of individual bias in the studies

The main problems among the articles were related to the lack of definition of the controls and the non-response rate (**Table 3**). All case-control studies scored well on the following items: “Is the case definition adequate?”, “Determination of exposure” and “Same verification method for cases and controls.” Regarding the “representativeness of the cases,” 4 articles (3, 15, 24, 25) did not describe the origin of the sample used. In the item “controls selection,” 5 studies (3, 15, 24) did not score because the origin of the control group is not mentioned. Major problems were identified in the “defining controls” domain, and only seven studies investigated the participants’ history of obesity. Most studies (3–5, 7, 12, 13, 18, 24, 25, 28, 29) used one or more parameters to compare groups; however, three studies (14, 15, 23) did not score in this regard. The Quality Assessment of studies included according to New Castle Ottawa protocol is exposed in **Tables 3**–**5**.

**Table 3:** Quality Assessment of Case-control studies included, according to New Castle Otawa protocol.

Evaluation for Case-Control Studies									
Authors (year)	Selection				Comparability	Exposure			
	Is the case definition adequate?	Representativeness of cases	Controls Selection	Defining Controls	Comparability of cases and controls based on design or analysis	Determination of exposure	Same verification method for cases and controls	Non-response rate	
**Miller et al., 2019** (1)	☆	☆	☆	☆	☆☆	☆	☆	☆	Good quality
**Karmous et al., 2018** (26)	☆	☆	☆	☆	☆☆	☆	☆	–	Good quality
**Park et al., 2015** (39)	☆	☆	☆	–	–	☆	☆	–	Fair quality
**Pepino et al., 2010** (35)	☆	–	–	–	☆	☆	☆	–	Poor quality
**Drewnowski, et. al., 1985** (29)	☆	☆	☆	–	–	☆	☆	–	Fair quality
**Thompson et al., 1977** (33)	☆	–	–	–	–	☆	☆	–	Poor quality
**Thompson et al., 1976** (32)	☆	–	–	☆	☆☆	☆	☆	–	Good quality
**Grinker et. al., 1972** (31)	☆	☆	☆	–	☆☆	☆	☆	–	Good quality
**Hardikar et al., 2017** (2)	☆	–	☆	☆	☆	☆	☆	–	Good quality
**Szalay et al., 2012** (37)	☆	☆	☆	☆	☆☆	☆	☆	☆	Good quality
**Vereczkei et al., 2011** (36)	☆	☆	☆	☆	☆	☆	☆	☆	Good Quality

" - ", it means no information / not informed / not applicable.

**Table 4:** Quality Assessment of cohort study included, according to New Castle Otawa protocol.

Evaluation for cohort studies									
Authors (year)	Selection				Comparability	Outcome			
	Representativeness of the exposed cohort	Selection of the Unexposed Cohort	Exposure determination	Demonstration that the outcome of interest was not present at baseline	Comparability of cohorts based on design and analysis	Determination of the outcome	Follow-up was long enough for results to occur.	Adequacy of cohort follow-up	
**Deglaire et. al., 2015** (25)	☆	☆	☆	☆	☆☆	☆	☆	☆	Good Quality

**Table 5:** Quality Assessment of Cross-Sectional studies included, according to New Castle Otawa protocol.

Evaluation for Cross-Sectional studies								
Authors (year)	Selection				Comparability	Outcome		
	Sample representativeness	Sample size	non-responders	Determination of exposure (risk factor)	Subjects in different outcome groups are comparable, based on study design or analysis. Confounding factors are controlled.	Assessment of outcome	Statistical test	
**Garcia et. al., 2017** (41)	☆	–	☆	☆	☆☆	☆☆	☆	Good quality
**Pepino and Mannella 2012** (35)	☆	–	☆	–	☆☆	☆	☆	Good quality
**Leohr et. al., 2020** (30)	☆	☆	☆	☆☆	☆☆	☆☆	☆	Good quality
**Tucker et. al., 2014** (10)	☆	☆	☆	☆☆	☆☆	☆☆	☆	Good quality
**Proserpio et al., 2016** (40)	☆	☆	☆	☆☆	☆☆	☆☆	☆	Good quality
**Malcolm et al., 1980** (27)	☆	–	☆	☆☆	☆☆	☆☆	–	Good Quality
**Snoek et al., 2004** (34)	☆	☆	☆	☆☆	☆☆	☆☆	–	Good Quality

" - ", it means no information / not informed / not applicable.

### 3.4 Certainty of evidence

Regarding the outcome on sweet tastes, 14 observational studies showed low certainty of evidence, indicating that the true effect may be substantially different from the estimated one. As for salty, bitter, and fatty flavors, the 13 observational studies showed moderate certainty of evidence. Regarding sour flavors, five observational studies showed high certainty of evidence, denoting high reliability that the actual effect is close to the effect estimate. **Table 6** summarizes these assessments.

**Table 6:** Summary of GRADE assessment of each outcome.

Obesity compared to non-obesity for food taste				
Patient or population: food taste Setting: Intervention: obesity Comparison: non-obesity				
Outcome N. of participants (studies)	Relative effect (95% CI)	Anticipated absolute effects (95% CI)	Certainty	What happens
Sweet taste			⨁⨁◯◯ Low^a^	The evidence suggests that obesity does not increase/reduce sweet taste.Three studies showed that obese participants had a higher threshold compared to non-obese for sweet taste. One study found that the non-obese group preferred lower concentrations of sweet fat and two studies observed that the obese group preferred higher concentrations of sweet.
N. of participants: 442 with obesity/ 501 without obesity	not estimable	not estimable		
(14 observational studies)				
Salty taste N. of participants: 128 with obesity/ 190 without obesity (5 observational studies)	not estimable	not estimable	⨁⨁⨁◯ Moderated^b^	The evidence suggests that obesity does not increase/reduce salty taste.Three studies showed that obese participants had a higher threshold compared to non-obese for salty taste. One study found that the obese group preferred higher concentrations of salt.
Bitter taste N. of participants: 177 with obesity/ 190 without obesity (4 observational studies)	not estimable	not estimable	⨁⨁⨁◯ Moderated^b^	The evidence suggests that obesity does not increase/reduce bitter taste. Two studies observed that people with normal weight had a higher threshold of perception for bitter taste, and three studies showed that obese participants had a higher threshold compared to non-obese.
Sour taste N. of participants: 69 with obesity/ 139 without obesity (5 observational studies)	not estimable	not estimable	⨁⨁⨁⨁ High	The evidence suggests that obesity does not increase/reduce sour taste. Two studies observed that people with normal weight had a higher threshold of perception for sour taste, and three studies showed that obese participants had a higher threshold compared to non-obese.
Fatty foods N. of participants: 108 with obesity/ 121 without obesity (4 observational studies)	not estimable	not estimable	⨁⨁⨁◯ Moderated^c^	The evidence suggests that obesity does not increase/reduce preferences for fatty foods. One study observed a greater response for the hedonic pleasure of obese in relation to fatty tastes.

CI, confidence interval.

GRADE Working Group grades of evidence.

High certainty: we are very confident that the true effect lies close to that of the estimate of the effect.

Moderate certainty: we are moderately confident in the effect estimate: the true effect is likely to be close to the estimate of the effect, but there is a possibility that it is substantially different.

Low certainty: our confidence in the effect estimate is limited: the true effect may be substantially different from the estimate of the effect.

Very low certainty: we have very little confidence in the effect estimate: the true effect is likely to be substantially different from the estimate of effect.

**Explanations:**

a. Three studies presented a fair quality and one study was categorized as presenting poor quality;

b. One study (39) presented fair quality;

c. One study (29) presented fair quality.

## 4 Discussion

This review aimed to gather evidence that would contribute to the scientific community’s understanding possible factors associated with obesity and new possible ways to aid in treating and diagnosing this pathology. Thus, we sought to assess the existence of an association between obesity and taste alterations, finding twenty articles, fifteen of them with good methodological quality and low risk of bias, four with medium risk, and three with low quality and high risk of bias. Among the selected studies, most test results supported the hypothesis of this association’s existence.

The human gustatory system allows the assessment of nutrition and toxicity during food consumption, which assists in the decision of what to eat (10). However, taste acts as an essential checkpoint and plays the most crucial role in the process of acceptance or rejection of food, preferences, and food options and, consequently, influences nutritional status and health (10). Although this association between taste acceptability and food choice has been determined, the extent of individual taste perception in relation to body weight is not well understood (36). Olfactory and gustatory sensations can induce pleasure, commonly associated with excessive food consumption, one of the leading causes of obesity (12). Greater intensities of sweet taste perception may induce stronger hedonic sensations, which may modulate eating behavior, leading to a preference for very sweet and energy-rich foods, indicating a possible etiological factor of obesity (29).

In this systematic review, four articles (26, 35, 39, 40) observed that participants with obesity had a higher threshold of gustatory perception; that is, people with obesity take longer to be able to recognize the taste of food, denoting that they are less sensitive in terms of taste. Studies show that obese or overweight people have a greater preference for sweeter and fatty foods (42, 43), which could be explained by the theory that obese people need higher concentrations of compounds responsible for flavoring food, thus justifying a greater consumption of salt and sugar, for example.

On the other hand, some studies (2, 10, 29–31, 35, 38) reported that participants with obesity felt the flavors at suprathreshold concentrations, that is, above the threshold of gustatory perception, equally intense to the control groups. That is, the ability to discriminate the presence of sweet and/or salty flavors in isomolar concentrations of salt and sugar was similar in both groups. Only one study (25) identified that people with obesity had a high intensity of gustatory perception, that is, the sweet and salty flavors were more intensely experienced, showing a greater gustatory sensitivity for this group. However, all of them had limitations since they used a model of sucrose, monosodium glutamate (MSG), or sodium chloride (NaCl) solution diluted in water at increasing concentrations, which may not be applicable to real life.

Brondel (24) cites the existence of sensory phenomena known to modulate the inhibition of food intake by reducing the pleasure derived from olfactory and gustatory stimuli in humans. These phenomena are directly associated with the feeling of satiety. They are divided into three mechanisms: conditioned satiety, alimentary alliesthesia, and sensory-specific satiety, the latter being investigated by several articles included in this review. According to their study (24), “sensory-specific satiety” is defined as “a relative decrease in the pleasure aroused by a food that has been consumed until satiety, in contrast to uneaten food.” Miller (1) states that the decrease in taste perception is not just a result of satiety and that marginal perceptions of taste seem to decrease due to sensory boredom resulting from repeated consumption of the same item. Thus, the continuous consumption of a type of food creates specific satiety and induces reduced consumption. In individuals without obesity, these sensory phenomena act as negative and final feedback mechanisms for food intake. Therefore, the dysfunction of these mechanisms can be one of the causes of excessive mass gain.

Regarding the outcome of specific sensory satiety, presented in two articles, one result (38) showed that, when consuming food repeatedly, the obese group took longer to experience sensory satiety specific to the food in question. This delay could justify a longer time of food consumption for people with obesity compared to people without obesity. However, another study (34) that evaluated this parameter did not identify differences between the groups. A specific article (1) observed that, in addition to the obesity group having presented higher initial levels of perception intensity than the control group, it also showed a lower perception decline speed than the control group, that is, the specific sensory satiety of the group with obesity manifested itself more slowly, which corroborates other results found (38). Therefore, to help create more effective and specific interventions in treating obesity, understanding the particularities of taste perception plays a vital role in elucidating new risk factors for obesity or obesity phenotype (1). However, the literature is still quite controversial regarding the influence of these phenomena on obesity.

Two studies (36, 37) evaluated the group’s brain activation concerning food consumption in a condition of the intrinsic physiological state of hunger and satiety maintained at a constant level. The neurophysiological investigation of the neural factors that may lead to obesity is an innovative and promising form of research since taste perception is microscopically initiated when food molecules interact with taste receptors existing on the surface of the cells that make up the taste buds, thus generating impulses to the central nervous system related to taste and promoting behavioral responses to different food stimuli in different aspects such as preference, threshold and supra-threshold sensitivity, hedonism, among others. Thus, the authors of both studies reported that, for the analyzes using the compounds: sucrose, vanilla, and quinine, participants with obesity showed greater brain activation in several areas related to taste perception, with emphasis on the hedonically positive stimulation of sucrose in the secondary gustatory cortex (COF) and in the cingulate cortex, both responsible for encoding the reward value of a particular flavor, showing that this may be a more accurate method in helping to understand the neural factors that can lead to obesity.

A case-control study (23) evaluated the association of the composition of the oral microbiota around contoured papillae and salivary parameters involved in sensitivity to oral fat with the threshold of detection of linoleic acid (fatty acid widely found in foods). This research was based on data that indicate that obesity seems to be associated with alterations in taste detection parameters, such as the flow of salivary activity or lipase activity. However, its results did not show differences between the groups with and without obesity.

Some articles included in this review have observed a greater sensitivity of the group with obesity to the salty and bitter taste in threshold concentrations (26, 35, 39, 40). Moreover, there was a preference for higher concentrations and higher hedonic responses (2, 25, 29, 35), especially for the sweet taste. However, the combined results of many studies have obtained inconclusive results, which have found no apparent difference in threshold and suprathreshold sensitivity and hedonic response between individuals with and without obesity (10, 10, 26, 27, 29–31, 35, 37, 38).

An additional interesting information is that Cabanac (13) theorized that food intake is physiologically monitored and regulated according to the needs of each individual, associating pleasure and body mass, thus as long as food contributes to the maintenance of predefined body weight by an individual set point inherent (“ponderostat”), the taste of that food will be perceived pleasantly, inducing a greater consumption, on the other hand, the additional ingestion of food beyond the necessity would make the taste of the food become unpleasant, avoiding the excess of food consumption (32). However, this theory has not been fully confirmed.

To qualify the methods used in the studies, the Newcastle-Otawa Scale was adopted. This protocol helped to assess the consistency and validity of the results generated by the observational studies included in this review. One study (33) presented more significant methodological problems (definition and selection of controls, representativeness of cases, comparability between them, and non-response rate), thus presenting a high risk of bias and low methodological quality. To make a more reliable analysis of the association between the multifactorial pathology in question and taste alterations, there should be a more significant control of confounding factors, using one or more comparability parameters between the groups, which was not done in the methodology of this study, in addition, the obesity history of the participants was also not investigated, as well as the origin of both the groups and the non-response rate, thus this study obtained the worst methodological rating among the others.

On the other hand, another case-control study (37) obtained the maximum score for methodological quality by investigating the groups’ brain activity in the face of gustatory stimulation. Three other cross-sectional studies also receive the maximum qualitative score, with five stars in the selection criterion, two in the comparability criterion, and three in the outcome, demonstrating excellent reliability in the results presented.

Most of the results expressed an association between obesity and taste alterations. On the other hand, the selected articles have some methodological limitations that directly affect the analyses presented here. One of the major limitation observed was the way obesity is diagnosed through BMI, which was used by most studies. More recent researches and guidelines indicate that this parameter, despite being frequently used in nutritional assessments, is very imprecise, since it doesn’t consider body composition, i.e., it doesn’t distinguish fat mass from muscle mass. Therefore, people with a lot of muscle mass can have a high BMI, even though they have a low percentage of body fat. Similarly, people with low muscle mass may have a BMI within the healthy range, but a high body fat percentage. Thus, more current and accurate methods of diagnosis is through analysis of body fat measurement, such as electoral bioimpedance or Dual-energy X-ray absorptiometry, which is not performed by most studies and would decrease the risk of methodological bias for that matter (44).

Another methodological deficiency is about the method for evaluating taste alterations, since many studies use only sucrose or sodium chloride solutions diluted in water at different concentrations to analyze hedonic response, preference and intensity (2, 27, 31–33, 38, 40) which is not representative of a normal daily diet, and therefore does not indicate results compatible with reality. In addition, all the studies are punctual evaluations, clippings of a continuous panorama that does not provide information on how long the patient has had an obesity condition and how the time factor may have influenced this scenario and consequently this association.

Futhermore, studies that despite presenting a well-designed, ethical, and thorough methodology, express some possible limitations that could interfere with the absence of statistical difference. To illustrate, the study from Leohr et al., 2020 (30) conducted a sugar/fat preference test (SFPT) after a standardized lunch meal to avoid any influence of hunger on the scoring of the solutions. However, it is possible that the meal itself could have affected the SFPT results. In addition, studies using only female participants (34, 35, 38) have obtained no statistical difference in some analyses, which could be explained by the fact that the study may be more susceptible to confounding variables that are related to gender, as hormonal differences and menstrual cycle phases can have a direct influence on women’s eating behavior, potentially impacting the results (44).

The heterogeneity and lack of standardization of methods among the included studies was a limiting factor regarding the possibility of making direct comparisons and making more accurate and safe inferences. Therefore, the lack of homogeneity prevented the performance of a meta-analysis in order to evaluate the real difference in perception, sensitivity, and taste pleasure between obese adults and people with average weight.

The studies found in this review aimed to elucidate possible physiological factors associated with the development of this complex pathology, such as behavioral phenotypes, unique brain activations, and distinct sensory responses. Nevertheless, due to the subjectivity of most analysis methods and the great variety of factors that could influence the results, many of the data obtained were inconclusive. Therefore, there is a need for more research on the possible factors associated with obesity and the necessity to create more accurate assessment methods.

Even though 40% of the included studies (2, 29–31, 33, 35, 38) reported that participants with obesity perceived flavors at suprathreshold concentrations (i.e. above the threshold of gustatory perception, with the same intensity as the control groups), methodological tests could be developed and applied in future studies to simulate conditions closer to reality and standardize the methods of measuring the perception of sensitivity to flavors.

## 5 Conclusion

According to the articles included, there is a possible association between obesity and taste alterations since most of them report some association between different taste alterations and this pathology. However, further longitudinal investigations using more sensitive methodologies are needed to describe the establishment of these alterations and their interactions with other factors.

## Data availability statement

The original contributions presented in the study are included in the article/supplementary material. Further inquiries can be directed to the corresponding author.

## Author contributions

BP, and RL: study concept and design. LB, BP, DF, MM, NF, LM, and RL: analysis and interpretation of data. BP, DF, MM, NF, MV, DS, LP, LM, and RL: preparation of the manuscript. LM, NF, MM, MV, LP, DS, and RL: critical revision of the manuscript. All authors contributed to the article and approved the submitted version.
